# Mesh *versus* suture repair of primary inguinal hernia in Ghana

**DOI:** 10.1002/bjs5.50186

**Published:** 2019-06-25

**Authors:** S. Tabiri, F. Owusu, F. Atindaana Abantanga, A. Moten, D. Nepogodiev, O. Omar, A. Bhangu

**Affiliations:** ^1^ School of Medicine and Health Sciences University for Development Studies Tamale Ghana; ^2^ Tamale Teaching Hospital Tamale Ghana; ^3^ St Patrick Hospital Offinso Ghana; ^4^ Department of Surgery Temple University Hospital Philadelphia Pennsylvania USA; ^5^ National Institute for Health Research Global Health Research Unit on Global Surgery, Institute of Translational Medicine University of Birmingham Birmingham UK

## Abstract

**Background:**

Most patients in Ghana undergo suture repair for primary inguinal hernia. Although there is strong evidence from high‐income country settings to indicate superiority of mesh repair for inguinal hernia, the evidence to support the safety and effectiveness of mesh repair in the Ghanaian setting is limited. This study aimed to compare hernia recurrence rates following suture *versus* mesh repair in Ghana.

**Methods:**

Men aged 18 years or over presenting with symptomatic, reducible inguinal hernias were included. Over the first 6 months all consecutive patients were enrolled prospectively and underwent a standardized suture repair; an equal number of patients were subsequently enrolled to undergo mesh repair. The primary outcome was hernia recurrence within 3 years of the index operation. Multivariable analysis was adjusted for age and right or left side. Adjusted odds ratios (ORs) with 95 per cent confidence intervals are reported.

**Results:**

A total of 116 sutured and 116 mesh inguinal hernia repairs were performed. Three years after surgery, follow‐up data were available for 206 of the 232 patients (88·8 per cent). Recurrence occurred significantly more frequently in the suture repair group (23 of 103, 22·3 per cent) than in the mesh group (7 of 103, 6·8 per cent) (*P* = 0·002). In multivariable analysis, suture repair was independently associated with an increased risk of recurrence (OR 4·51, 95 per cent c.i. 1·76 to 11·52; *P* = 0·002).

**Conclusion:**

In Ghana, mesh inguinal hernia repair was associated with reduced 3‐year recurrence compared with sutured repair. Controlled dissemination across Ghana should now be assessed.

## Introduction

**Figure 1:**
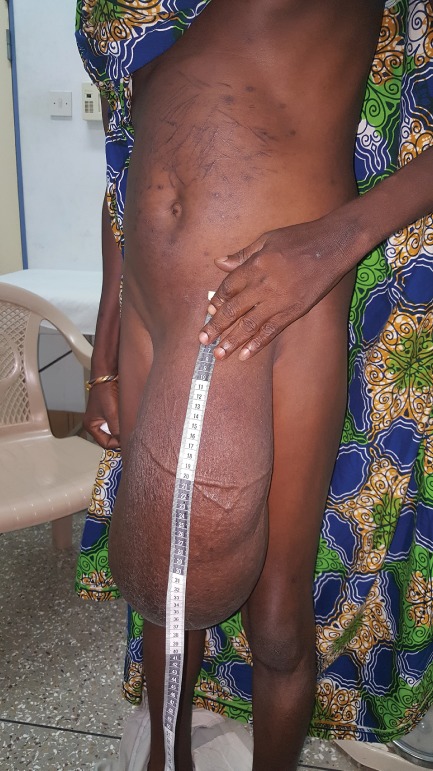
Large inguinoscrotal hernia

Recent population‐based studies from sub‐Saharan Africa have indicated a prevalence of inguinal hernia in adult men of 7–13 per cent, with a high frequency of inguinoscrotal hernia (*Fig*. 1)^1^, ^2^, ^3^. Inguinal hernia repair is the second most common procedure performed in district hospitals in Ghana^4^. The current surgical repair rate of 123 per 100 000 population in Northern Ghana is insufficient to meet demand^5^. It is estimated^6^ that by 2022 there will be one million people in Ghana with an inguinal hernia in need of surgical repair. This high burden of inguinal hernia means it is a priority condition in Ghana.

Compared with sutured repair, Lichtenstein tension‐free inguinal hernia repair is superior, with a lower recurrence rate in high‐income countries^7^, ^8^. In Ghana, although the majority of patients are able to access hernia surgery free of charge through the National Health Insurance Scheme, the cost of mesh is not covered. Across sub‐Saharan Africa, most patients are unable to pay out of pocket to receive standard commercial mesh^9^. Consequently, most hernia repairs in Ghana are sutured^5^. Although the cost of suture repair for hernia is low compared with treatment of infectious diseases such tuberculosis, human immunodeficiency virus/acquired immune deficiency syndrome and malaria^2^, health authorities will remain unlikely to dedicate additional resources to purchase mesh unless there is evidence of safety and efficacy in the African setting.

In collaboration with the Ghana Health Service, the Ghana Hernia Society has prioritized increasing the availability of mesh inguinal hernia repair. This study aimed to determine the safety and efficacy of a change in practice from suture to mesh repair in a small district hospital in Ghana.

## Methods

**Figure 2:**
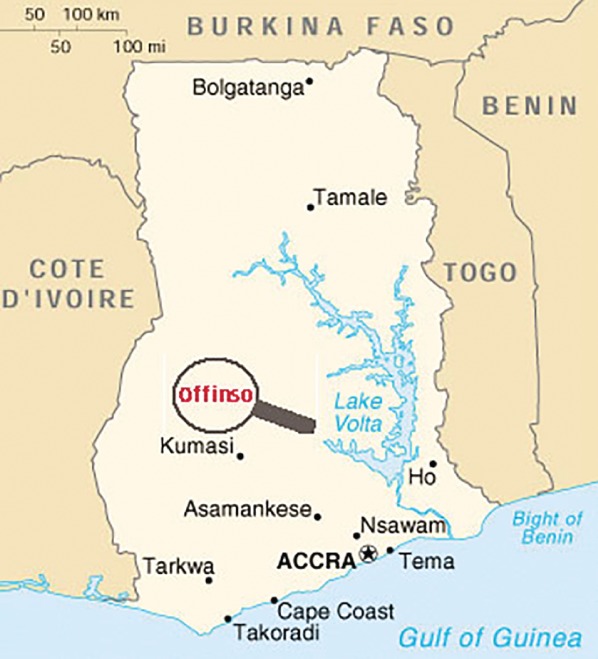
Location of St Patrick Hospital, Offinso, Ghana

This study was conducted at St Patrick Hospital, a 120‐bed district hospital in Offinso, Ghana (*Fig*. 2). The hospital is located in the Ashanti Region in one of the transition zones between Southern and Northern Ghana. The district's population in the 2010 census was 56 881, with most people being subsistence farmers^10^. The hospital staff includes one general surgeon, who performs all the hernia repairs.

This study was approved by the St Patrick Hospital administration and Tamale Teaching Hospital ethical review committee (reference TTHERC/17/11/16/09). Patients were consented before surgery for participation in the study, including additional clinical follow‐up. The use of mesh repair for inguinal hernia has approval from the Ghana Health Service and the Ministry of Health, Ghana.

### Study design

Consecutive men aged 18 years or more with a symptomatic, reducible inguinal hernia (Kingsnorth H1–H3 classification^11^) were included. If a patient had bilateral inguinal hernias, the side that the patient felt had the most severe symptoms was repaired. All patients initially presented to the outpatient clinic for examination and investigation, and were booked for elective inguinal hernia repair.

To avoid selection bias, the study was conducted in two phases. The first phase lasted 6 months (1 February 2014 to 30 July 2014), and during this period all eligible patients were identified and consented, and had a suture repair of the hernia (suture repair group). The second phase (1 August 2014 to 1 January 2015) ran until an equal number of consecutive patients had been identified and consented as in the first phase. All patients in the second phase underwent mesh repair of the hernia (mesh repair group). Patients were blinded to the repair technique. All procedures were undertaken by a single surgeon. Before commencement of the study this surgeon was fully competent in both suture and mesh repair, having completed over 200 previous hernia repairs.

### Surgical technique

Suture repair was a modified Bassini technique. The transversalis fascia and the conjoint tendon were sutured to the inguinal ligament behind the spermatic cord with a monofilament non‐absorbable suture. A vertical relaxing incision was made in the anterior rectus to reduce tension on the suture line (Tanner slide).

For the mesh repair, a monofilament polypropylene flat mesh (BARD® Mesh; C.R. Bard, Warwick, Rhode Island, USA) was fixed to the inguinal ligament with a non‐absorbable suture and to the conjoint tendon with an absorbable suture. The mesh was provided to patients free of charge during the study. All operations were performed under spinal anaesthesia using bupivacaine (3–3·5 ml; 15–17·5 mg).

### Outcome measures

The primary outcome measure was hernia recurrence at 3 years after surgery. Recurrence was defined as the presence of a visible or palpable expansile cough impulse at the hernia orifice of the site of surgery on clinical examination.

Secondary outcome measures included reoperation, mortality, haematoma, surgical‐site infection and acute pain (all at 14 days), and chronic pain at 2 years after surgery. Chronic pain was defined as any pain or unpleasant sensory and emotional experience associated with actual or potential tissue damage, or described in terms of such damage that persisted beyond the normal healing period of 22 weeks by the International Association for the Study of Pain^12^.

### Outcome assessment

Patients were invited to attend an outpatient clinic at the hospital at 2 weeks, 1 year, 2 years and 3 years after surgery. If patients did not attend, they were followed up in the community with a home visit. Follow‐up was performed by a medical officer who had not been involved in the surgery, blinded to the surgical technique used for each patient. The medical officer had completed medical school and house officer postgraduate training, and was trained in a standard examination technique to assess for inguinal hernia. Patients were examined standing up. The hernia orifices were palpated and the patient was then asked to cough in order to detect any cough impulse.

### Statistical analysis

Data were held in a password‐protected database on a secure computer in a locked office by one of the authors. Continuous data are reported as mean(s.d.) values, and Student's *t* test was used to compare differences between groups. Categorical data are reported as percentages; the χ^2^ test was used to compare differences between groups. A multivariable logistic regression model was used to adjust recurrence for patient age and the side of hernia repair. Odds ratios (ORs) are reported with 95 per cent confidence intervals. *P* < 0·050 was considered to indicate statistical significance for all tests. Statistical analysis was performed in SPSS® version 22 (IBM, Armonk, New York, USA).

## Results

A total of 116 men were identified and underwent sutured inguinal hernia repair. A further 116 were recruited to the mesh repair group. The mean age of patients was 45·4(19·1) years in the suture phase and 42·0(15·1) years in the mesh phase (*P* = 0·126) (*Table* 1).

**Table 1:** Demographics and outcomes

	Mesh repair (*n* = 116)	Suture repair (*n* = 116)	*P* ‡
**Age (years)** *	42·0(15·1)	45·4(19·1)	0·126§
**Laterality**			0·892
Right	72 (62·1)	73 (62·9)	
Left	44 (37·9)	43 (37·1)	
**Early complications** †			
Haematoma	2 (1·7)	1 (0·9)	0·561
SSI	0 (0)	1 (0·9)	0·316
Pain	13 (11·2)	10 (8·6)	0·510
None	101 (87·1)	104 (89·7)	0·539
**Chronic pain at 2 years**			0·225
No	103 of 108 (95·4)	111 of 113 (98·2)	
Yes	5 of 108 (4·6)	2 of 113 (1·8)	
No follow‐up	8	3	
**Recurrent hernia at 3 years**			0·002
No	96 of 103 (93·2)	80 of 103 (77·7)	
Yes	7 of 103 (6·8)	23 of 103 (22·3)	
No follow‐up	13	13	

Values in parentheses are percentages unless indicated otherwise;

* values are mean(s.d.).

† At 14 days after surgery. SSI, surgical‐site infection.

‡ χ^2^ test, except

§ Student's *t* test.

There were no reoperations or deaths at 14 days after surgery, and there were no differences in haematoma, surgical‐site infection or acute pain rate at 14 days (*Table* 1). Data on chronic pain up to 2 years after surgery were available for 221 of the 232 patients (95·3 per cent). The overall chronic pain rate was 3·2 per cent (7 of 221). The rate was slightly higher in the mesh group than in the suture group (4·6 *versus* 1·8 per cent respectively), but this difference was not statistically significant (*P* = 0·225).

At 3 years after surgery, follow‐up was completed for 206 of the 232 patients (88·8 per cent). The overall 3‐year recurrence rate was 14·6 per cent (30 of 206). Recurrence occurred significantly more frequently in the suture repair than in the mesh repair group (22·3 *versus* 6·8 per cent respectively; *P* = 0·002) (*Table* 1). In multivariable analysis, suture repair was independently associated with increased odds of recurrence (OR 4·51, 95 per cent c.i. 1·76 to 11·52; *P* = 0·002) (*Table* 2).

**Table 2:** Multivariable model for hernia recurrence up to 3 years after surgery

	Univariable analysis	Multivariable analysis	
	Odds ratio	Odds ratio	*P*
**Repair method**			
Mesh	1·00 (reference)	1·00 (reference)	
Suture	3·94 (1·61, 9·66)	4·51 (1·76, 11·52)	0·002
**Age**	0·96 (0·93, 0·99)	0·96 (0·93, 0·98)	0·002
**Laterality**			
Right	1·00 (reference)	1·00 (reference)	
Left	3·05 (1·38, 6·76)	3·34 (1·42, 7·85)	0·006

Values in parentheses are 95 per cent confidence intervals.

## Discussion

This study found that mesh inguinal hernia repair was associated with significantly fewer hernia recurrences. Mesh repair was safe with no increase in complications at either 14 days or 2 years. These findings are consistent reports^8^, ^13^ that have demonstrated that mesh repair reduces recurrence rates by half or more. The study also found no increase in chronic pain following mesh inguinal hernia repair compared with suture repair, as noted in previous RCTs^13^, ^14^, ^15^.

Around 85 per cent of inguinal hernia repairs in Ghana are performed using a suture technique^5^. Patients are more likely to undergo mesh repair if their surgery is performed by a surgeon rather than a medical officer who has not completed formal surgical training, or the operation is performed at a teaching hospital *versus* a district hospital^5^. A key barrier to mesh repair in Ghana has been the cost of mesh, with commercial mesh costing up to €100 per unit^16^. Although some studies found mosquito net mesh to be a safe and cost‐effective alternative to commercial mesh, mosquito mesh is no longer in routine use as the supply of suitable mesh is unreliable^16^, ^17^, ^18^, ^19^.

A limitation of this study is that only one surgeon was available to perform hernia repairs at the participating centre. These results may not be generalizable across Ghana. However, by standardizing surgical technique, this single‐surgeon series reduced potential bias that might result from variable surgical technique if multiple surgeons had participated. Risk of bias was reduced by robust assessment of hernia recurrence involving a standardized examination technique performed by a trained medical officer with patients blinded to surgical technique.

By 2022, approximately one million people in Ghana will have an inguinal hernia in need of surgical repair^6^, so dissemination in Ghana of a cost‐effective method of hernia repair that minimizes the risk of recurrence is essential. The process should be monitored as mesh inguinal hernia repair is rolled out across Ghana. As most inguinal hernia repairs in Ghana are performed by non‐surgeon physicians^5^, programmes should be developed to train these practitioners in mesh repair. Evaluation of task‐shifting in this context is likely to be influential in informing the scale‐up of surgical capacity by healthcare providers in other low‐ and middle‐income countries.
